# A Multivariate Age-Structured Stochastic Model with Immunization Strategies to Describe Bronchiolitis Dynamics

**DOI:** 10.3390/ijerph18147607

**Published:** 2021-07-17

**Authors:** Mónica López-Lacort, Ana Corberán-Vallet, Francisco J. Santonja Gómez

**Affiliations:** 1 Vaccine Research Department, Fisabio-Public Health, Avda. Cataluña 21, 46020 Valencia, Spain; monica.lopez@fisabio.es; 2 Department of Statistics and Operations Research, Universitat de València, Dr. Moliner 50, 46100 Burjassot, Spain; Francisco.Santonja@uv.es

**Keywords:** infectious diseases, bronchiolitis, respiratory syncytial virus, stochastic Bayesian model, multivariate age-structured model, immunization programs

## Abstract

Bronchiolitis has a high morbidity in children under 2 years old. Respiratory syncytial virus (RSV) is the most common pathogen causing the disease. At present, there is only a costly humanized monoclonal RSV-specific antibody to prevent RSV. However, different immunization strategies are being developed. Hence, evaluation and comparison of their impact is important for policymakers. The analysis of the disease with a Bayesian stochastic compartmental model provided an improved and more natural description of its dynamics. However, the consideration of different age groups is still needed, since disease transmission greatly varies with age. In this work, we propose a multivariate age-structured stochastic model to understand bronchiolitis dynamics in children younger than 2 years of age considering high-quality data from the Valencia health system integrated database. Our modeling approach combines ideas from compartmental models and Bayesian hierarchical Poisson models in a novel way. Finally, we develop an extension of the model that simulates the effect of potential newborn immunization scenarios on the burden of disease. We provide an app tool that estimates the expected reduction in bronchiolitis episodes for a range of different values of uptake and effectiveness.

## 1. Background

Bronchiolitis is a common lower respiratory tract infection (LRTI) that mainly affects children under 2 years old, with the greatest burden occurring in infants younger than 6 months [1]. Respiratory syncytial virus (RSV) is by far the most common cause of viral bronchiolitis [2]. Previous data from the Valencia region of Spain showed that approximately 2 out of 10 children younger than 2 years of age are diagnosed of bronchiolitis, 3 out of 100 are hospitalized and 1.6 out of 100 are hospitalized with RSV bronchiolitis [3,4].

At present, palivizumab is the only prophylactic therapy available for RSV. This is a costly humanized monoclonal RSV-specific antibody that is given monthly to infants at increased risk of severe RSV infection: premature babies, infants with chronic lung disease and infants with congenital heart disease [5,6]. However, different RSV immunization strategies, including maternal immunization, immunoprophylaxis with new monoclonal antibodies (mAbs) and pediatric immunization, are currently under development and could be available soon [7,8].

Immaturity of the infant’s immune system does not allow active immunization until 2 months of age. However, this population subgroup is more susceptible to severe RSV infection. Therefore, passive immunization practices (maternal vaccination and mAbs administered at birth) have the potential to be the most effective strategies to protect individuals at an early age. ResVax is the most advanced maternal vaccine in clinical development. In a phase III multi-country, randomized, placebo-controlled trial evaluating its efficacy against RSV-LRTI in infants from birth to 90–180 days of life, ResVax showed 44% efficacy in reducing RSV-LRTI hospitalization. However, it failed to meet the primary outcome of prevention of medically significant LRTI [9]. A phase III trial showed the potential of mAb nirsevimab to protect infants for an entire RSV season with just one injection. A single dose of this mAb with an extended half-life resulted in a lower incidence of medically attended RSV-associated LRTI and hospitalizations than with a placebo in healthy preterm infants entering their first RSV season. They were approximately 70% and 80% lower, respectively [10].

Before the licensure of these products for RSV and the implementation of a particular preventive strategy, policymakers will need to assess their potential health and economic benefits. This requires an exhaustive evaluation of the real burden of disease and the simulation of disease incidence under different immunization scenarios.

Different studies have been proposed to analyze either bronchiolitis or RSV dynamics. Most of these studies consider classical regression models to describe causal relationships between risk factors (baseline characteristics or clinical features) and disease counts [4,11,12,13,14,15]. Deterministic compartmental models based on differential equations have also been commonly implemented to model RSV dynamics [16,17,18,19]. Deterministic models assume that every individual has an equal probability of contacting every other individual in the population, so they may fail if the sizes of the compartments are not large enough to ensure homogeneous mixing. The incorporation of stochasticity into epidemic models is necessary for considering heterogeneous mixing of individuals in the population and also the inherent stochasticity in the transmission of disease [20,21,22,23]. A Bayesian stochastic compartmental model in discrete time has improved the description of RSV disease [24].

Alternative stochastic approaches for the analysis of time series of infectious disease counts have been described by Held et al. [25]. They explain disease counts by means of a Poisson (or negative binomial) model with two components: a parameter-driven component that describes endemic seasonal patterns and an observation-driven component, which is an autoregression on past counts that explains localized epidemics. This model can be extended in a multivariate setting to analyze time series that correspond to disease counts in different spatial units or different age groups jointly [26].

Systematic reviews of model-based evaluations of immunization strategies against RSV have been presented in [27,28]. Many of these studies use mathematical models based on ordinary differential equations (see, for instance, [17,29,30]). Bayesian stochastic compartmental models have also been extended to simulate and assess the effect of a vaccination strategy that consists of vaccinating a proportion of newborns [31]. Since clinical data are not yet available, models exploring the epidemiological and economic outcomes of potential RSV interventions adopt different assumptions regarding the target population, uptake rates, effectiveness and duration of immunity. Therefore, the estimates that are obtained vary considerably.

In this paper, we present a Bayesian multivariate age-structured model in discrete time to describe bronchiolitis dynamics in the region of Valencia in a population of children under 2 years of age. In particular, the population is divided into four age groups, and new infections are described considering interaction among these groups. We base our formulation on a Poisson model (or a negative binomial model if the data exhibit overdispersion). The main innovation is that the mean of the distribution at each time point can be seen as an observation-driven component where the autoregressive parameter varies stochastically over time. A parameter allowing for heterogeneous mixing of individuals in the population is also introduced to capture different contact patterns. This modeling approach does not require information on susceptibles, and it also provides a simplified framework to describe disease counts, since it avoids the modeling of complex transitions between the different compartments. To estimate the impact of potential prevention strategies for RSV, an extension of the model including simulation of bronchiolitis episodes under different passive immunization scenarios is considered.

## 2. Data

### 2.1. Population of Interest

The region of Valencia, one of the 17 autonomous regions of Spain, has approximately 4,900,000 inhabitants. Around 2% of the Valencia population is younger than 2 years old (approximately 100,000 children). The Regional Health System (RHS) is divided into 241 health care districts structured into 24 health departments. It includes 34 public hospitals, 24 of them attending acute pediatric patients. This study includes children under the age of 2 years who were born in the region of Valencia between January 2009 and December 2012 and are covered by the RHS.

### 2.2. *Data Sources*

The region of Valencia has a health system integrated database (VID) that gathers health and sociodemographic data from 98% of the population [32]. In particular, we used the population information system (an administrative database that collects and updates sociodemographic data from both residents and non-residents with access to public health services) to determine the population of interest. Primary care electronic medical notes (SIA) were implemented in 2006, and all medical visits are registered and ICD-coded. Hospitalizations were obtained using discharge reports from the Spanish Minimum Basic Data Set (MBDS).

### 2.3. *Age-Structured Bronchiolitis Cases*

Because around 90% of bronchiolitis cases in children less than 2 years of age are treated in primary care offices and very few cases have a RSV microbiological confirmation, the analysis of laboratory-confirmed cases of RSV bronchiolitis is less useful for incidence estimation due to underreporting. Hence, it is more practical to analyze bronchiolitis-associated outpatient visits and hospitalizations to evaluate the potential impact of RSV immunization strategies.

In particular, we analyze bronchiolitis episodes identified from hospitalization and primary care attendance through a search of the first appearance of the following ICD-9-codes 466.1, 466.11 and 466.19, in MBDS and SIA. Based on the McConnochie criterion, only the first health care encounter (either outpatient visit or hospitalization) with a bronchiolitis ICD-9-MC code was counted as a case of bronchiolitis.

**Figure 1:**
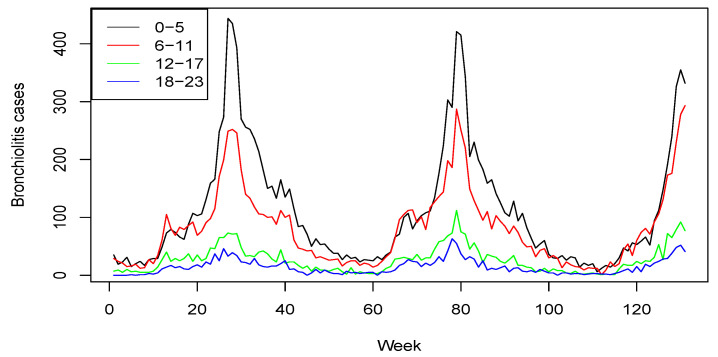
Weekly counts of bronchiolitis for the four age groups from July 2010 (week 28 June to 4 July) to December 2012 (week 24 December to 30 December).

Considering that children aged less than 6 months have an elevated morbidity, we consider four age groups: 0–5, 6–11, 12–17 and 18–23 months of age. This grouping allows us to analyze in greater detail both transmission dynamics and the impact of potential immunization measures. Weekly aggregated cases from July 2010 (week 28 June–4 July) to December 2012 (week 24 December–30 December) are considered (T = 131 weeks in total). Since this is a population-based cohort study, data for all the age groups were not available until July 2010, so data from January 2009 to June 2010 are excluded in the analysis. In total, the study includes 30,555 cases of bronchiolitis: 14,635 (47.90%) of them are in children aged less than 6 months, 10,600 (34.69%) cases in the age group of 6–11 months, 3462 (11.33%) in the age group of 12–17 months and 1858 (6.08%) in the age group of 18–23 months. Figure 1 shows the time plot of the series for the four age groups. As can be seen, there is a higher incidence for younger children and a clear seasonal pattern, with more cases during the weeks of autumn and winter. These data can be downloaded from https://rotapp.shinyapps.io/APP_IMPACTO_RSV, (accessed on 14 May 2021).

## 3. Model Description

### 3.1. Background

The most commonly used models in the analysis of infectious disease counts are compartmental models, which divide the population being studied into different compartments according to disease status and describe the evolution of infection through changes in the number of individuals in each compartment. Corberán et al. [24] proposed a Bayesian stochastic compartmental model in discrete time to describe RSV dynamics in the Region of Valencia. Let $y_{t},t=1,2,\cdots ,T,$ be the number of newly infected individuals at week *t*. The model assumes that:

(1) $$y_{t}\;∼\;Bi\left(S_{t-1},p_{t}\right),$$

where $S_{t-1}$ is the susceptible population at time t−1, which is updated weekly using some recursion equations, and $p_{t}$ is the probability of becoming infected at time *t*. To take into account the transmissible nature of the infection, $p_{t}$ is modeled as:

(2) $$p_{t}=\frac{y_{t-1}^{h}\cdot \exp\left(r_{t}\right)}{1+\;y_{t-1}^{h}\cdot \exp\left(r_{t}\right)},$$

where the mixing parameter *h* allows for heterogeneous mixing of individuals in the population and $r_{t}=\alpha_{0}+\sum_{w=1}^{W}\left(\beta_{w}\cdot \sin\left(\frac{2\cdot \pi \cdot w\cdot t}{52}\right)+\;\gamma_{w}\cdot \cos\left(\frac{2\cdot \pi \cdot w\cdot t}{52}\right)\right)+\varepsilon_{t}$. By allowing the transmission rate $\exp\left(r_{t}\right)$ to vary over time, the stochastic model provided an improved and accurate description of the pattern of disease.

When the focus is on describing counts of new infections, the use of a Bayesian hierarchical model provides an alternative framework. Held et al. [25] proposed a stochastic model for the analysis of disease counts based on a Poisson (or negative binomial) model with two components, which describe endemic seasonal patterns and localized epidemics. The starting point was a simple branching process model, which was later extended to include seasonal terms in the endemic rate or to adjust for overdispersion. In particular, weekly new counts of disease $y_{t},t=1,2,\cdots ,T,$ are modeled as:

(3) $$y_{t}∼\;Poisson\left(\nu_{t}+\lambda_{t}\cdot y_{t-1}\right),$$

where $\nu_{t}$ is the parameter-driven (or endemic) component and $\lambda_{t}\cdot y_{t-1}$ the observation-driven (or epidemic) component, which allows for occasional outbreaks. A main feature of the proposed model is that the autoregressive parameter λ is allowed to vary over time. To do this, a Bayesian changepoint model with an unknown number of changepoints is used to capture sudden changes in infectiousness. The endemic component is described as follows:

(4) $$\nu_{t}=\exp\left(\alpha_{0}+\beta \cdot \sin\left(\frac{2\cdot \pi \cdot t}{52}\right)+\gamma \cdot \cos\left(\frac{2\cdot \pi \cdot t}{52}\right)\right).$$

Multivariate extensions of that model can be found in [26] for the joint analysis of multiple time series of counts, where each component corresponds to a geographical region or a certain age group. Let $y_{t}^{j}$ denote the count of disease in age group *j* at week *t*. In the multivariate scenario, counts of disease can be described as:

$$\begin{array}{ccc} y_{t}^{j} & ∼ & \;Poisson\left(\nu_{t}^{j}+\lambda \cdot y_{t-1}^{j}\right),\\ \nu_{t}^{j} & = & \exp\left(\alpha_{0}^{j}+\alpha_{1}\cdot t+\sum_{w=1}^{W}\left(\beta_{w}\cdot \sin\left(\frac{2\cdot \pi \cdot w\cdot t}{52}\right)+\gamma_{w}\cdot \cos\left(\frac{2\cdot \pi \cdot w\cdot t}{52}\right)\right)\right), \end{array}$$

where parameters $\alpha_{0}^{j}$ allow for different incidence levels in the different age groups. The epidemic component here depends only on previous counts in the corresponding age group.

A more general model considering previous counts in other age groups as potential explanatory variables is formulated as:

$$y_{t}^{j}∼\;Poisson\left(\nu_{t}^{j}+\lambda \cdot y_{t-1}^{j}+\phi \cdot \sum_{i\neq j}y_{t-1}^{i}\right),$$

where the additional parameter ϕ captures the autoregressive effect of the other age groups. Paul et al. [33] extended this multivariate model by allowing the autoregressive parameters to depend on the age-group; that is, $\lambda^{j}$ and $\phi^{j}$ for each time series. However, in these multivariate models, the autoregressive parameters λ and ϕ are not allowed to vary over time.

### 3.2. Our Proposal

Let $y_{t}^{j}$ denote the count of disease in age group j,j=1,2,3,4, observed at week *t*, t=1,2,⋯, T=131. We use a Bayesian hierarchical model, which provides a straightforward framework to describe counts of new infections at each week. Our model assumes that the probability governing the counts is a Poisson distribution or a negative binomial distribution if the data exhibit overdispersion. In those situations in which the population size is not large enough in comparison with the observed counts of disease, the binomial distribution could be used in a similar way.

Seasonal variation in disease transmission that is persistent with a stable pattern is modeled through sine–cosine waves, which are common for all groups. This is a sensible assumption since annual epidemic peaks occur simultaneously in the different age groups. On the other hand, it is reasonable to assume that contacts among the different age groups considered here are equally likely to occur. Hence, counts of disease at time t−1 are summed up to describe new infections. In addition, heterogeneous mixing of individuals in the population is allowed. Finally, due to the epidemiology of bronchiolitis, it is important to consider a different transmission rate for each age group.

Taking into account these considerations, the proposed model is given by the following equations:

(5)ytj∼Poissonμtj,(6)μtj=exp(λtj)·∑i=14yt−1ih,(7)λtj=α0j+β·sin2·π·t52+ γ·cos2·π·t52+εt,

where the autoregressive parameter $\exp(\lambda_{t}^{j})$ is allowed to vary stochastically over time by means of random effects $\left\{\varepsilon_{t}\;∼\;N\left(0,\sigma_{\varepsilon }^{2}\right)\right\}$ that represent unspecified features of week *t*. To avoid overfitting, these random effects are common for all the age groups; that is, if counts of disease at a particular week are higher (or lower) than expected for some age groups, that increase (or decrease) should also be observed in the remaining age groups. h∈[0,1] is the mixing parameter (h=1 would correspond to the assumption of mass action). Non-informative flat prior distributions are considered for parameters $\alpha_{0}^{j}$, β and γ. The uniform distribution in the interval [0,1] is considered as a prior for *h*, and the uniform distribution in the interval (0,2) is considered for the standard deviation $\sigma_{\varepsilon }.$

To adjust for overdispersion, the negative binomial distribution can be used instead. In that case, the model can be formulated as

(8)ytj∼NegBin(ptj,k),(9)ptj=kk+μtj,

where $\mu_{t}^{j}$ is defined as in Equation (6). Hence, the mean of the distribution is equal to $\mu_{t}^{j}$ and the variance is given by $\mu_{t}^{j}+\frac{{\left(\mu_{t}^{j}\right)}^{2}}{k}$. The second parameter k>0 incorporates the extra-Poisson variation (the limiting case k=∞ corresponds to the Poisson distribution). As a prior distribution for parameter *k*, we consider the Gamma distribution Ga(1,0.01).

It is important to emphasize that our formulation can be seen as a Poisson model with only one component, the observation-driven component [25,26], modified in a novel way to allow for contact heterogeneity. By considering a time-varying autoregressive parameter, the model properly describes both endemic an epidemic periods. As shown in the next section, this formulation will allow us to simulate disease incidence in a scenario with immunization measures. On the other hand, the mean of the distribution is equivalent to that proposed in [24], where the process intensity mean was dependent on the product of $S_{t-1}$, $y_{t-1}^{h}$ and the transmission rate $\exp\left(r_{t}\right)$. In our modeling framework, where information on susceptibles is not incorporated, the autoregressive parameter $\exp(\lambda_{t}^{j})$ describes how infected individuals in the previous week produce new infections.

### 3.3. Our Extension with Immunization Strategies

We show here how the proposed model can be used to simulate the impact of passive immunization through maternal vaccination or mAb administered at birth. The main parameters to consider when estimating the effects of a particular immunization strategy are the uptake, the efficacy and the duration of protection.

Based on vaccination coverage data provided by the Ministry of Health, Consumer Affairs and Social Welfare of Spain (https://www.mscbs.gob.es/profesionales/saludPublica/prevPromocion/vacunaciones/calendario-y-coberturas/coberturas/home.htm, accesed on 14 May 2021) and the clinical trial results presented in [9,10], the following assumptions are incorporated into the simulation process:

In the case of maternal vaccination:

1. A range of vaccine uptake from 50 to 85%;
2. 40% efficacy in reducing RSV bronchiolitis episodes;
3. Duration of protection of 6 months.

With respect to the use of mAb:

1. The uptake parameter can be considered to be 95%;
2. 70% efficacy in reducing RSV bronchiolitis episodes;
3. 6 months of induced immunity.

Because the duration of protection is assumed to be 6 months, children are immune from birth to 6 months, when they move to the next age group. Hence, in our modeling scheme, the evaluation of these two strategies come down to a decrease in the number of cases in the first age group (0–5 months) throughout the simulation study. Since we analyze total counts of bronchiolitis, it is also important to take into account that approximately 70% of bronchiolitis cases are due to RSV.

Table 1 shows the percentage of children in the age group 0–5 months that should be removed according to the specified values for the uptake and the efficacy parameters. These values are also in accordance with the assumptions made in some of the studies discussed in [27].

Let $\pi_{\mathrm{rsv}}$ represent the proportion of bronchiolitis cases due to RSV and $\pi_{\mathrm{im}}$ the proportion of children in the age group 0–5 months that are immune as a result of the implementation of an immunization strategy. For age group 1 (0–5 months), the number of infected children at week *t* can be estimated from our model as:

(10) $${\hat{y}}_{t}^{1}=\left(1-\pi_{\mathrm{im}}\right)\cdot \pi_{\mathrm{rsv}}\cdot \left(\exp\left(\lambda_{t}^{1}\right)\cdot {\left(\sum_{i=1}^{4}{\hat{y}}_{t-1}^{i}\right)}^{h}\right)+\left(1-\pi_{\mathrm{rsv}}\right)\cdot \left(\exp\left(\lambda_{t}^{1}\right)\cdot {\left(\sum_{i=1}^{4}{\hat{y}}_{t-1}^{i}\right)}^{h}\right);$$

that is, a proportion $\pi_{\mathrm{im}}$ is removed from the number of RSV bronchiolitis cases that would be expected from contacts with infected children at week t−1. Note that, under the immunization scenario, contacts of immune children will not end in contagion.

For the age groups j=2,3,4, infected children at week *t* can be estimated as:

(11) $${\hat{y}}_{t}^{j}=\exp\left(\lambda_{t}^{j}\right)\cdot {\left(\sum_{i=1}^{4}{\hat{y}}_{t-1}^{i}\right)}^{h}.$$

In the previous equations, $\exp\left(\lambda_{t}^{j}\right)\cdot {\left(\sum_{i=1}^{4}{\hat{y}}_{t-1}^{i}\right)}^{h}$ represents the mean $\mu_{t}^{j}$ of the distribution governing the counts, which is either the Poisson or the negative binomial distribution, based on the simulated counts under the immunization scenario. Parameters $\left\{\exp\left(\lambda_{t}^{j}\right)\right\}$ and *h* are, respectively, the autoregressive parameters and the mixing parameter of the model without a immunization strategy (see Equation (6)). It is important to emphasize that these parameters represent features of bronchiolitis dynamics (how the disease spreads at each time point for the different age groups) and contact patterns that do not depend on the number of infected children in previous weeks. Hence, they can be used to simulate the number of infections after a particular preventive intervention has been implemented.

## 4. Results

R Statistical Software (Foundation for Statistical Computing, Vienna, Austria) and WinBUGS program [34] were used to perform the analysis using MCMC simulation methods. A total of 25,000 iterations were used as the burn-in period of the MCMC. After that, 75,000 iterations were run, and only 1 in every 150 of them was kept to reduce correlation. Two chains were simulated, so M=1000 values were simulated in total from the posterior distribution. MCMC convergence was assessed by visual inspection of the trace plots, the Brooks–Gelman–Rubin scale reduction factor (Rhat equal to 1 means good convergence) and the effective sample size (n.eff above 100 means good convergence). All statistical analyses of the study are completely reproducible. The BUGS code used can be found as supplementary material to the paper.

### 4.1. Results without Immunization Strategies

**Figure 2:**
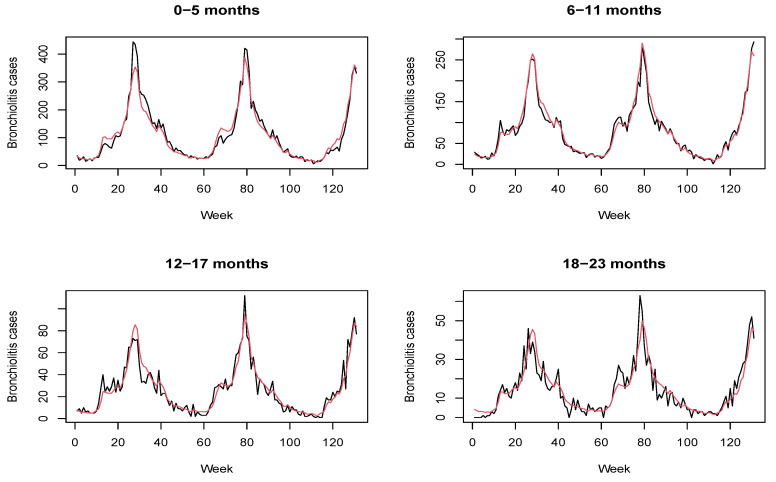
Model accuracy. Bronchiolitis counts (black line) together with posterior mean estimates (red line) from July 2010 to December 2012 (T = 131 weeks) obtained with the proposed model based on the negative binomial distribution.

We fitted both the Poisson and the binomial negative model to the bronchiolitis data. The corresponding DIC [35] values are, respectively, 3811.77 (pD 105.89) and 3710.97 (pD 83.96), so adjusting for overdispersion provides a better fit. The posterior mean of parameter *k* is 44.45, which is the 95% credible interval [32.65,59.41]. Figure 2 displays the posterior mean bronchiolitis temporal profile (red line) together with real counts of disease (black line). As can be seen, the proposed model is able to accurately recover disease dynamics in all the age groups.

**Figure 3:**
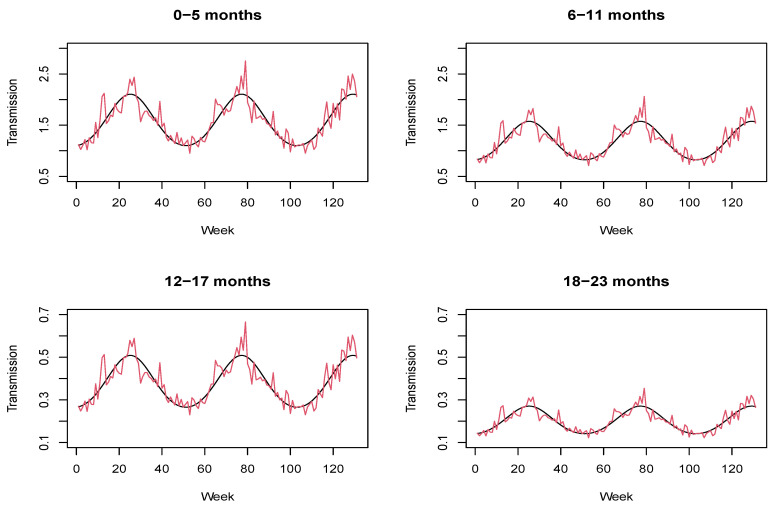
Estimated autoregressive parameter (red line) together with its seasonal component (black line).

Figure 3 shows the estimated autoregressive parameter $\exp\left(\lambda_{t}^{j}\right)$ in each age group and time point together with its seasonal component, which is given by:

$$\exp\left(\alpha_{0}^{j}+\beta \cdot \sin\left(\frac{2\cdot \pi \cdot t}{52}\right)+\;\gamma \cdot \cos\left(\frac{2\cdot \pi \cdot t}{52}\right)\right).$$

As expected, previous counts of disease have a higher impact on the youngest children. In fact, the transmission of disease decreases as the age increases. This figure also demonstrates that seasonality has a strong influence on transmission dynamics. However, because the seasonal pattern varies slightly from year to year, the incorporation of random effects accounting for stochasticity in the transmission is fundamental to providing a more accurate description of the data. These results agree with those obtained in [24]. In that paper, the proposed model with a stochastic transmission rate led to an improved goodness of fit in comparison with its counterpart where the seasonal pattern repeated over time; that is, there were no weekly random effects in the model assumed for the transmission rate.

#### Model Comparison

In order to assess the performance of our model, we also analyzed the bronchiolitis data with a Poisson model whose mean is given by the sum of an endemic and an epidemic components. Taking into account the assumptions made in this particular case study (common seasonality for all ages and contacts among the different age groups that are equally likely to occur), the Poisson model with two components is formulated as:

(12)ytj∼Poissonνtj+λj·∑i=14yt−1i(13)νtj=expα0j+β·sin2·π·t52+ γ·cos2·π·t52.

To ensure that the mean of the Poisson is non-negative, parameter $\lambda^{j}$ is modeled as $\lambda^{j}=\exp\left(\delta^{j}\right)$, with $\delta^{j}∼N\left(0,\sigma_{\delta }\right).$

**Figure 4:**
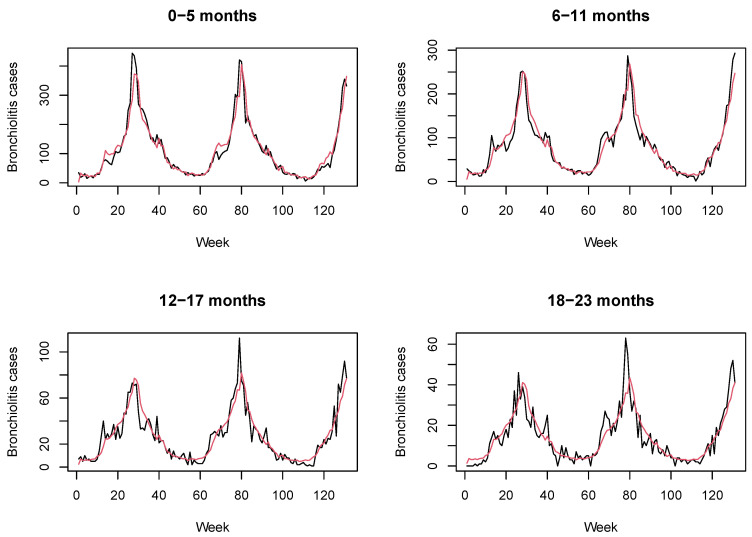
Bronchiolitis counts (black line) together with posterior mean estimates (red line) obtained with a Poisson model with two components.

Figure 4 displays the posterior mean bronchiolitis temporal profile (red line) corresponding to this Poisson model with two components together with real counts of disease (black line). The DIC value for this model is 4506.18 (pD 9.88).

This two-component model is able to describe the overall dynamics of bronchiolitis. However, the Poisson model proposed here provides a better fit, as judged by a lower DIC (3811.77 against 4506.18). This may be due to the fact that the epidemic component, which depends on a time-constant autoregressive parameter, cannot explain the stochasticity inherent in transmission dynamics.

### 4.2. Results with Immunization Strategies

Once we have obtained a sample from the posterior distribution of the parameters of our model without a immunization program, we can evaluate the effect of the immunization strategies previously described. We assume here that the immunization strategy started six months before the first week of July 2010 (week 28 June to 4 July), so that the specified percentage (see Table 1) of children in the age group of 0–5 months can be assumed to be immune at time t=1 of our analysis. We simulate the evolution of bronchiolitis counts using Equations (10) and (11), assuming $\pi_{\mathrm{rsv}}=0.7$. Note that for each iteration of the MCMC simulation, we have one simulated value of the regression parameters and the mixing parameter $\left\{\exp\left(\lambda_{t}^{j(m)}\right)\right\}$ and $h^{\left(m\right)}$, m=1,2,…,M=1000, so we have *M* simulated values for the number of infections for each age group and week. Using these values, we can get point estimates (for instance, the mean of the simulated values) as well as credible intervals.

**Figure 5:**
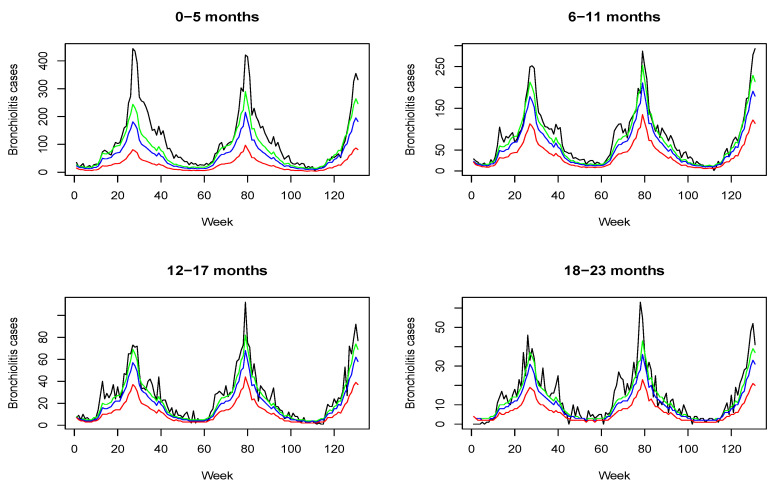
Bronchiolitis counts (black line) together with simulated counts (mean values) for three different immunization proportions: $\pi_{\mathrm{im}}$ = 0.2 (green line), 0.34 (blue line) and 0.67 (red line).

Figure 5 shows real counts of bronchiolitis and point estimates (mean values) for three simulation scenarios, corresponding to $\pi_{\mathrm{im}}$ = 0.2 (green line), 0.34 (blue line) and 0.67 (red line). As expected, the number of bronchiolitis cases decreases as the proportion of immune children in the age group of 0–5 month increases. Because of herd protection, the decrease can be observed in all the age groups. The decrease expected in each age group and the total decrease are shown in Table 2.

We also developed an R Shiny application (https://rotapp.shinyapps.io/APP_IMPACTO_RSV, accessed on 14 May 2021) that simulates the evolution of bronchiolitis that would be obtained by varying the uptake and the efficacy parameters, as well as the percentage of bronchiolitis cases due to RSV.

## 5. Conclusions

We have developed an age-structured stochastic model that allows us to accurately explain bronchiolitis dynamics in the region of Valencia. Our modeling approach combines ideas from compartmental models and Bayesian hierarchical Poisson models in a novel way. By using a hierarchical Poisson (or a binomial negative) model, we do not require information on susceptibles and we avoid the modeling of complex transitions between the multiple compartments. Unlike standard formulations, the main innovation of our model is that the mean of the distribution at each time point depends only on an observation-driven component where the autoregressive parameter is allowed to vary stochastically over time. A mixing parameter is also introduced to capture heterogeneous mixing of individuals in the population. By modeling the dynamics of disease as a function of previous counts, we can simulate disease incidence under different immunization scenarios. Note that the incorporation of a parameter-driven component would hinder simulation of disease evolution when a decrease in the number of cases at previous weeks has been obtained as a result of the assumed immunity.

Even though we have developed the model for the analysis of bronchiolitis, it can be adapted for other infectious diseases with (or without) a seasonal pattern, replacing the transmission rate according to the nature of disease.

The extension proposed in this paper provides a useful framework to address one of the important needs in RSV bronchiolitis incidence control: the implementation of an immunization strategy. We have assessed here the effects of passive immunization through maternal vaccination or mAb administered at birth. In our modelling scheme, the evaluation of these two strategies come down to a decrease in the number of cases in the first age group (0–5 months) throughout the simulation study. We have simulated the evolution of bronchiolitis counts for different values of the uptake and efficacy. The duration of immunity has been assumed to be equal to 6 months. If immunity lasts more than 6 months, the model can be adapted so that a percentage of children in the age group of 6–11 months is also removed, since children would move to this age group being immune for a certain number of weeks. On the other hand, if immunity lasts less than 6 months, the percentage of children removed in the age group 0–5 months should be reduced accordingly.

The results obtained show that newborn immunization contributes to a substantial decrease in the number of bronchiolitis infections. Because of herd immunity, this decrease is also observed in all the age groups considered. As expected, the decrease is greater when the uptake and/or the efficacy increase. As the profiles of the products under development become better defined, further alignment will be possible.

We have developed an R Shiny application that simulates the evolution of bronchiolitis episodes for different values of the uptake and efficacy, as well as the percentage of bronchiolitis cases due to RSV. This application is an easy tool that allows users and decision makers to interact with the simulation analysis and improves visualization of the results.

We have not assessed here the impact of immunization of high-risk children, such as preterm infants or children with high-risk comorbid conditions. It may be important to consider a separate analysis for these groups most at risk.

Another fruitful area for further research would be the consideration of smaller age groups so that different immunization strategies such as vaccination of infants older than 3 months could also be tested. Stratification by gestational age may also be important when evaluating maternal vaccination strategies, since transfer of maternal antibodies for preterm infants may be incomplete.

## Figures and Tables

**Table 1:** Possible scenarios in the evaluation of immunization strategies.

Maternal Vaccination			mAb at Birth		
**Uptake**	**Efficacy**	**% Removed**	**Uptake**	**Efficacy**	**% Removed**
50%	40%	20%	95%	70%	66.50%
85%	40%	34%			

**Table 2:** Expected decrease in the number of bronchiolitis counts for three different immunization proportions: $\pi_{\mathrm{im}}$ = 0.2, 0.34 and 0.67.

$\pi_{\mathrm{im}}$	0–5 Months	6–11 Months	12–17 Months	18–23 Months	Overall
0.2	28.94%	14.63%	15.48%	16.25%	21.68%
0.34	47.48%	28.75%	29.78%	30.03%	37.92%
0.67	76.32%	54.17%	54.56%	54.84%	64.87%

## Data Availability

Not applicable.

## Supplementary Materials

The following are available online at https://www.mdpi.com/article/10.3390/ijerph18147607/s1.
